# Development of the gut microbiota during early life in premature and term infants

**DOI:** 10.1186/s13099-022-00529-6

**Published:** 2023-01-16

**Authors:** Kathleen Sim, Elizabeth Powell, Emma Cornwell, J. Simon Kroll, Alexander G. Shaw

**Affiliations:** 1 Section of Paediatric Infectious Disease, Department of Infectious Disease, Imperial College London, London, W2 1PG UK; 2 Department of Infectious Disease Epidemiology, School of Public Health, Imperial College London, Sir Michael Uren Building, 84 Wood Lane, London, W12 0BZ UK

**Keywords:** Microbiota, Neonatal, Development, Premature infant, Term infant, Atopy

## Abstract

**Background:**

The gastrointestinal (GI) microbiota has been linked to health consequences throughout life, from early life illnesses (e.g. sepsis and necrotising enterocolitis) to lifelong chronic conditions such as obesity and inflammatory bowel disease. It has also been observed that events in early life can lead to shifts in the microbiota, with some of these changes having been documented to persist into adulthood. A particularly extreme example of a divergent early GI microbiota occurs in premature neonates, who display a very different GI community to term infants. Certain characteristic patterns have been associated with negative health outcomes during the neonatal period, and these patterns may prove to have continual damaging effects if not resolved.

**Results:**

In this study we compared a set of premature infants with a paired set of term infants (n = 37 pairs) at 6 weeks of age and at 2 years of age. In the samples taken at 6 weeks of age we found microbial communities differing in both diversity and specific bacterial groups between the two infant cohorts. We identified clinical factors associated with over-abundance of potentially pathogenic organisms (e.g. *Enterobacteriaceae*) and reduced abundances of some beneficial organisms (e.g. *Bifidobacterium*). We contrasted these findings with samples taken at 2 years of age, which indicated that despite a very different initial gut microbiota, the two infant groups converged to a similar, more adult-like state. We identified clinical factors, including both prematurity and delivery method, which remain associated with components of the gut microbiota. Both clinical factors and microbial characteristics are compared to the occurrence of childhood wheeze and eczema, revealing associations between components of the GI microbiota and the development of these allergic conditions.

**Conclusions:**

The faecal microbiota differs greatly between infants born at term and those born prematurely during early life, yet it converges over time. Despite this, early clinical factors remain significantly associated with the abundance of some bacterial groups at 2 years of age. Given the associations made between health conditions and the microbiota, factors that alter the makeup of the gut microbiota, and potentially its trajectory through life, could have important lifelong consequences.

**Supplementary Information:**

The online version contains supplementary material available at 10.1186/s13099-022-00529-6.

## Background

The gut microbiota is a vast reservoir of bacteria living within a human host with interactions both beneficial and harmful. The community plays an important role in the digestion of food, the development of the immune system and in the maturation of the gut itself [1–3]. By the point of birth, the gut has been seeded with an array of organisms that colonize and grow, only to be replaced by a succession of species as the environment changes [4, 5]. Great variety is seen in the diversity and abundance of organisms, and the patterns present have been associated with health conditions throughout life [6]. The causes of variation within the community are therefore of great interest in trying to predict—and potentially enhance—future health, and it has been indicated that factors in early life, such as mode of delivery, can still be reflected and observed in the patterns of the gut microbiota in adults [7].

We may therefore be concerned by the particularly extreme case of gut microbiota alteration that occurs as a result of the net effects of being born premature. These infants are found to have a drastically altered gut microbiota when compared to infants born at term [8, 9]. Prior research has demonstrated that particular community patterns are associated with conditions such as NEC and sepsis in early life, as well as conditions such as inflammatory bowel disease (IBD) and obesity in later life [4, 10–13]. The trajectory of the infant gut microbiota is therefore of great interest, with potential life-long consequences.

A particular area of interest for the infant microbiota is the potential relationship with allergic disease. The rapid rise in allergic disease including asthma and eczema may have an origin in the changing exposure to the environmental microbiota in recent years [14]. Environmental biodiversity and the individual’s early microbiota may affect the development of immune tolerance [14], and a critical window when the microbiota may exert its influence has been supported by both animal models [15] and cohort studies [16, 17]. Reduced diversity of the faecal microbiota has been associated with allergic sensitization [18], rhinitis [18] and later asthma [17] though not uniformly in all studies. Specific bacterial taxa present early in the faecal microbiota such as *Clostridium* have been associated with an asthma diagnosis [19] and allergic sensitization [20], and conversely reduction in other taxa such as *Lachnospira* have been associated with asthma; with this association confirmed in a mouse model [16].

In this study we sought to determine if the gut microbiota of term and premature infant cohorts converge by 2 years of age, and to study whether microbial signatures that differ between the cohorts are associated with wheezing and eczema.

## Methods

### Study population and sample collection

#### Preterm cohort

Premature infants (< 32 weeks gestation) who were admitted to an Imperial College Healthcare NHS Trust neonatal intensive care unit (NICU) (St Mary’s Hospital, Queen Charlotte’s and Chelsea Hospital) between January 2011 and December 2012 were recruited to the Neonatal Microbiota (NeoM) Study. The study was approved by the West London Research Ethics Committee Two, United Kingdom (reference number: 10/H0711/39). Parents gave written informed consent for their infant to join the study. Detailed daily clinical data was collected during the duration of the admission. We aimed to collect every faecal sample produced by each participant from recruitment until discharge. Nursing staff collected the samples from the nappies using a sterile spatula and placed them into a sterile DNase-, RNase-free microcentrifuge tube. Samples were stored at − 20 °C within two hours of collection and transferred to a − 80 °C freezer within 5 days, where they remained until DNA extraction.

Parents were re-approached when the participants were between the ages of 2 and 4 years to join the follow-up study (NeoM2) (approved by the London—Chelsea Research Ethics Committee—reference number: 13/LO/0693). Parents who consented to their child participating in the study completed a general health questionnaire for their child which contained questions specifically relating to allergic disease and wheezing episodes. A faecal sample from the child was also collected by the parents using a sterile scoop and placed into a sterile container which was then posted to the research laboratory within 24 h. Faecal samples were immediately stored at − 80 °C on receipt, where they remained until DNA extraction.

#### Term cohort

Expectant parents were approached in the antenatal clinics of St Mary’s hospital and asked for their assent to be approached when their baby was born. Subsequently, if the babies met the inclusion criteria (≥ 37 weeks gestation with no airway abnormalities and born at St Mary’s hospital), parents were re-visited on the postnatal ward or at home and their written informed consent was asked for their baby’s participation in the Development Of the Respiratory Microbiota (DORMIce) study. A smaller proportion was recruited directly on the postnatal ward. The study was approved by the London Riverside Research Ethics Committee, United Kingdom (Reference number: 12/LO/1362).

The study involved regular visits to the infants in the community at 6 weeks, 6, 9, 12, 18 and 24 months, and in a subset at 3 and 4.5 months. Faecal samples were collected at birth and at each timepoint by the parents from the nappy using a sterile scoop, placed into a sterile container and stored at room temperature. This was transported by the researcher to the laboratory or alternatively posted by the parents and subsequently frozen at − 80 °C. Mailed samples were stored an average 2 days after collection. Over this time period spanning collection and delivery we have not found any significant shifts in the microbiota of infant stool samples [21].

For each cohort, a sample closest to 6 weeks after birth and one taken at 2 years of age were chosen for analysis. Clinical data describing the infant’s feeds, living environment and health history was collected at each of the timepoints either by reference to clinical notes or through a questionnaire. For term infants at 2 years of age, parental consent was sought to contact GPs for a copy of the participant’s primary care record. Notes were reviewed for medical diagnoses including wheezing and eczema, antibiotics prescribed and immunisation record. Eleven infants across the combined cohorts were missing at least one point of clinical data. The assembled data for the term and premature infant cohorts is summarised in Table 1.

**Table 1:** Demographics of the infant cohorts at birth, 6 weeks and 2 years of chronological age

	Term	Premature
Birth demographics		
Female	19 (51.4)	14 (37.8)
Median birth weight (gram)	3510 (3130, 3690)	960 (765, 1325)
Mean gestation at birth (weeks + days)	40 + 2 (39 + 4 to 41 + 2)	28 + 1 (25 + 4 to 29 + 6)
Ethnicity		
Black	2 (5.4)	4 (10.8)
White	20 (54.1)	18 (48.4)
Asian	4 (10.8)	9 (24.3)
Mixed race	6 (16.2)	3 (8.1)
Other	5 (13.5)	3 (8.1)
Mode of delivery		
Caesarean section	8 (21.6)	8 (21.6)
Vaginal	29 (78.4)	29 (78.4)
Six week demographics		
Feeding at time of sampling		
Breast milk	26 (70.3)	26 (70.3)
Formula	10 (27.0)	10 (27.0)
Mixed	1 (2.7)	1 (2.7)
Number of complete months of breast milk feeds	1 (1, 1)	1 (0, 1)
Number of courses of antibiotics	0 (0, 0)	1 (1, 2)
Two year demographics		
Infant age at sampling (months)	24 (23.9, 24.2)	35.7 (31.1, 39.1)
Feeding at time of sample		
Weaned	37 (37, 37)	37 (37, 37)
Number of complete months of breast milk feeds	11 (7, 17)	6 (3, 9)
Age that the infant received non-breast milk feeds (months)	0 (0, 4)	3 (0, 6)
Number of courses of antibiotics since 6 weeks of age	1 (0, 2)	3 (0, 4)
Antibiotics use in the last month (yes)	2 (5.4)	2 (5.4)
Pet in the household (yes)	7 (18.9)	10 (27.0)
Other child in the household (yes)	15 (40.5)	21 (56.8)
Smoker in the household (yes)	5 (13.5)	2 (5.4)
Child suffered from one or more wheezing episodes (yes, parent reported)	18 (48.6)	13 (35.1)
Child suffers from eczema (yes, parent reported)	24 (64.9)	7 (18.9)

For continuous factors median and interquartile range are shown. Categorical factors are expressed in absolute value and percentage of the cohort in brackets

### Matching criteria

Thirty-seven infants from the NeoM study were matched to 37 infants from the DORMICe study by mode of delivery and the type of feeds (breast milk/formula/mixed feeds) that the infant was on at the time of collection of the 6 week faecal sample. Where possible, infants were also matched on maternal intrapartum antibiotic use and neonatal antibiotic use at birth, followed by ethnicity.

### Bacterial DNA extraction

DNA was extracted from the faecal samples (200 mg) using the FastDNA SPIN Kit for Soil (MP Biomedicals, Santa Ana, California), following the manufacturer’s protocol (inclusive of bead-beating homogenisation steps) except that the final elution step was into Tris (10 mM) low-ethylenediaminetetraacetic acid (0.1mM) buffer. DNA concentration and quality was confirmed using a NanoDrop Spectrophotometer, and the DNA was stored at − 80 °C prior to PCR.

### Amplification and pyrosequencing of the V3–V5 regions of the bacterial *16S rRNA* gene

Primers containing a unique 12-bp Golay barcode [22, 23] were used to amplify the V3–V5 region of the bacterial *16S rRNA* gene from each DNA sample. Amplicons were produced by polymerase chain reaction (PCR) as described previously [24]. The resulting pooled replicate amplicons were purified and three pyrosequencing runs were conducted on a 454 Life Sciences GS FLX (Roche) machine in accordance to the Roche Amplicon Lib-L protocol. Non-target controls were included to identify contamination.

### Data processing

Sequencing reads were analysed using the Quantitative Insights Into Microbial Ecology (QIIME) version 1.9.0 package [25], following the recommended pipeline for the combination of multiple 454 FLX datasets. Denoising was performed using denoise_wrapper.py, and the datasets integrated. Chimera removal was performed with ChimeraSlayer [26] and singletons removed. An average of 1657 sequencing reads was obtained per sample (see Additional file 1: Fig. S1 for read counts by sample). Sequences were clustered at 97% sequence identity using the UCLUST algorithm into operational taxonomic units (OTU) [27] and aligned to the SILVA rRNA database version 119 [28]. Rarefaction to 664 reads per sample was performed, removing heterogeneity of sequencing reads per sample whilst still retaining an accurate representation of diversity (see Additional file 1: Fig. S2). Diversity calculations were performed in the R statistical package [29] using vegan (Community Ecology Package: Ordination, Diversity and Dissimilarities) [30].

### Statistics

Statistical analyses were performed with the R statistical package [29]. Alpha diversity measures (Shannon Index, Shannon’s Equitability, the Inverse Simpson Index and Faith’s Phylogenetic Diversity) and Beta diversity measures (the Jaccard index, Bray-Curtis dissimilarity, unweighted unifrac and weighted unifrac) were calculated using QIIME [25]. Beta diversity distances between sample groups were compared using the Mann–Whitney U test (testing distances within groups to between groups) and the Wilcoxon signed-rank test (testing matched sets of distances at different time points). Alpha diversity was compared using the Wilcoxon signed-rank test. Canonical ordination analysis (CCA) was performed in R with the vegan statistical package [30].

Differentially abundant OTUs/phyla at 6 weeks and 2 years of age were detected using general linear models (GLM) with a negative binomial distribution, with weeks gestation at birth used as the explanatory variable and day of sampling added as a confounding factor. Multiple hypothesis corrections (MHC) were made with the Benjamini and Hochberg Procedure. All GLMs were performed using absolute numbers of rarefied sequencing reads.

Associations between clinical factors and OTUs/phyla were tested using GLMs with the clinical factor and as the explanatory variable and weeks gestation at birth and day of sampling added as confounding factors. MHCs were made with the Benjamini and Hochberg Procedure, and factors that were significant at 20% were carried forward. Where multiple factors were found to be significantly associated with OTUs/phyla, multivariate models were used. Models were then refined by sequential removal of the least significant factors until only factors significant at 5% remained. OTUs/phyla that are significantly associated with clinical factors through this method are documented in Additional file 1: Tables S3, S4, S7 and S8. Gestation at birth and day of sampling were retained in each model, with gestation at birth being documented if it significantly influenced the model.

Associations between eczema/wheeze occurrence and clinical factors and OTUs/phyla were tested using logistic regression models, again including gestation at birth and day of sampling added as confounding factors. Corrections were performed as for the GLM models, and univariate and multivariate models were processed in the same manner, although gestation at birth was only retained in the final multivariate model if found to provide significant improvement. The significance of retained factors was confirmed through likelihood ratio tests. Receiver Operating characteristics Curve (ROC) curves and area under the curves (AUC) were calculated using R. Validation of the models was performed using sets of 30 term and 30 premature infants drawn randomly from a pool of 64 premature and 35 term infants whose data was not used elsewhere in this manuscript. Infants were drawn from the pool 1000 times and the median ROC curve and inter-quartile range (IQR) calculated.

## Results

**Fig. 1:**
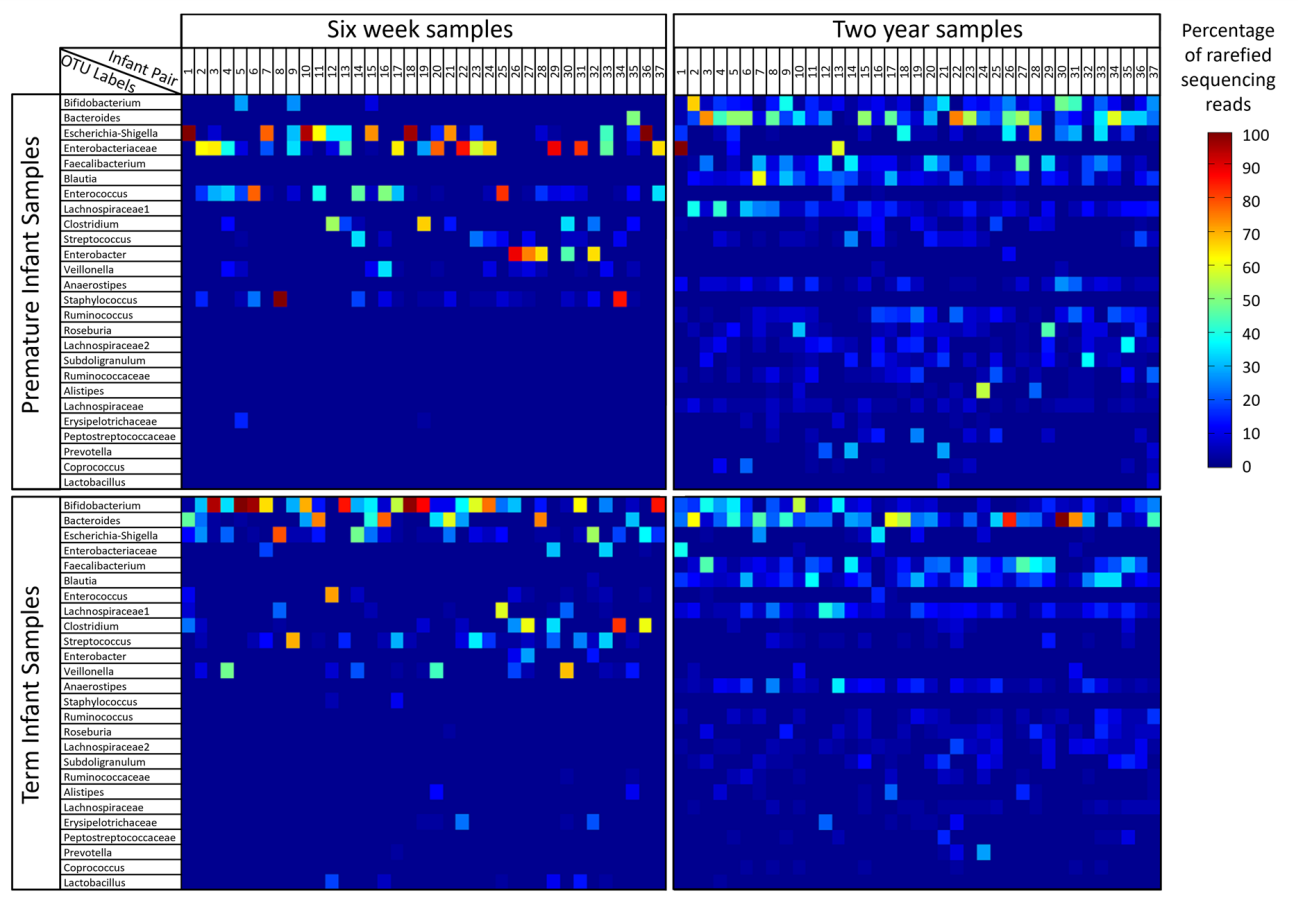
Heatmaps of rarefied sequencing data. The dataset is split into four panels according to the infant birth category (premature or term delivery) and the timepoint at which it was taken (6 weeks or 2 years). The distribution of sequencing reads for each sample is represented by a single column in one of the four panels, with colours indicating the percentage of reads assigned to each of the OTUs shown on the y axis. Within each panel, samples are organised along with x axis by the infant pair number. Comparing columns of data vertically therefore contrasts a premature and term infant pair of samples at a single timepoint, whilst comparison of columns of data horizontally between each panel allows assessment of the changes in an infant’s faecal microbiota from 6 weeks to 2 years of age

The study dataset comprised 37 infant pairs (one term and one premature infant) each with sequencing data from samples taken at 6 weeks and 2 years of age. Sequencing reads assigned to the 26 most abundant OTUS (making up 95% of the total reads in the dataset) are shown segregated by term/premature birth status and by timepoint in Fig. 1.

### The faecal microbiota of premature and term infants are significantly different at 6 weeks of age

**Fig. 2:**
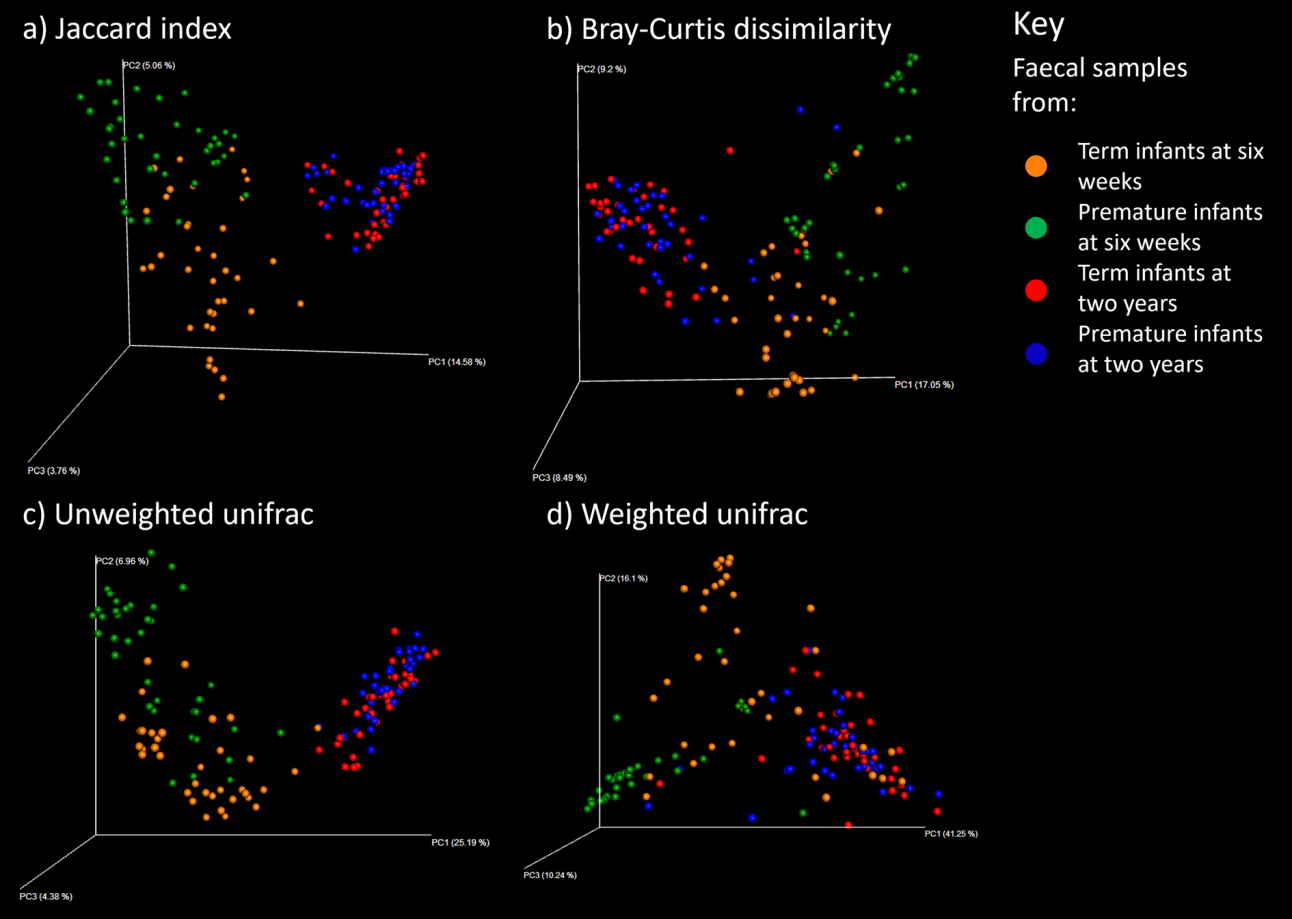
Measures of distances between samples displayed by principal coordinate analysis. Samples are colour coded according to the key

To determine how similar the sample groups were, distances between samples were calculated using four measures of beta diversity. These distances were plotted in principal coordinate analyses (see Fig. 2) which demonstrate clear separation of the premature and term infant faecal microbial communities at 6 weeks of age by each of the four measures.

**Fig. 3:**
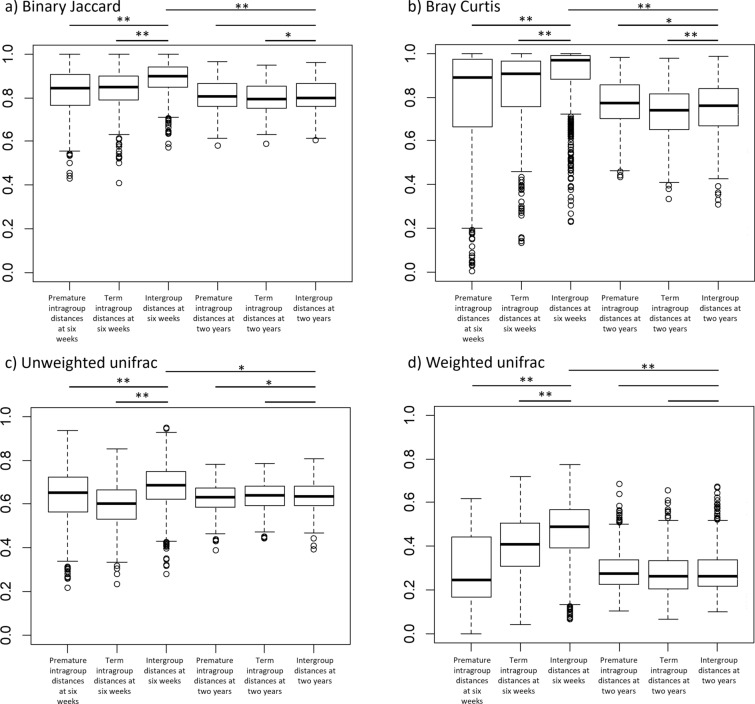
Comparisons of similarity between sample groups using four distance measures. Four sample groups have been analysed; samples from premature infants at 6 weeks of age, samples from premature infants at 2 years, samples from term infants at 6 weeks of age and samples from term infants at 2 years of age. Boxes indicate the 25% and 75% quartiles with the bar showing the median. Whiskers mark the quartiles ± 1.5 IQR. The x axis indicates the set of samples for which distances have been measured. Bars along the top of the charts indicate where a comparison has been made, either using a Mann Whitney U test (intragroup to intergroup distances) or a Wilcoxon signed-rank test (intragroup distances at 6 weeks to intragroup distances at 2 years of age) has been made. Where statistical tests found a significant difference, asterisks indicate the p value: * indicates 0.05 > p ≥ 0.001, ** indicates p < 0.001

The distances between samples within the groups were found to be significantly lower than the inter-group distances (see Fig. 3), indicating that there are significant sources of dissimilarity between the microbiota of term and premature infants at 6 weeks of age.

### Both diversity measures and specific OTUs differ between premature and term infant faecal microbial communities

Given the significant differences between the faecal microbial communities of premature and term infants at 6 weeks of age, we sought to characterise which traits of the community drove the separation.

**Fig. 4:**
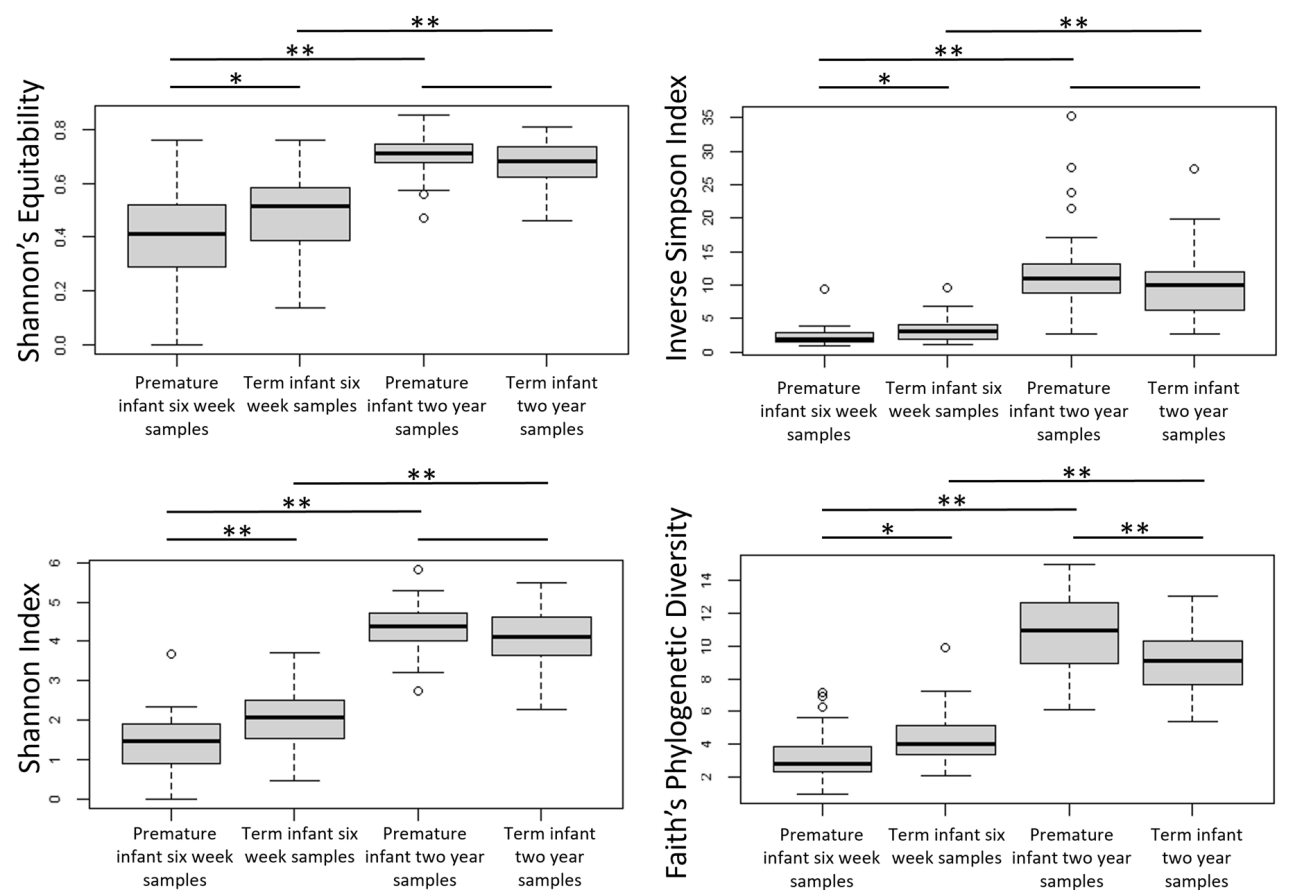
Diversity of the faecal microbial community in four groups of samples; as measured by the Shannon’s Equitability, the inverse Simpson Index and the Shannon Index. Boxes indicate the 25% and 75% quartiles with the bar showing the median. Whiskers mark the quartiles ± 1.5 IQR. Alpha diversity of sample groups was compared using the Wilcoxon signed-rank test. Where significant difference in diversity measures were found, asterisks indicate the p value: * indicates 0.05 > p = > 0.001, ** indicates p < 0.001

Four alpha diversity measures were calculated for each sample, with the results shown in Fig. 4. Comparisons between premature and term infant faecal samples at 6 weeks of age indicated a significant decrease in bacterial diversity in the premature samples by all four alpha diversity measures, reflecting a community that is less rich, less even and with lower genetic diversity.

**Fig. 5:**
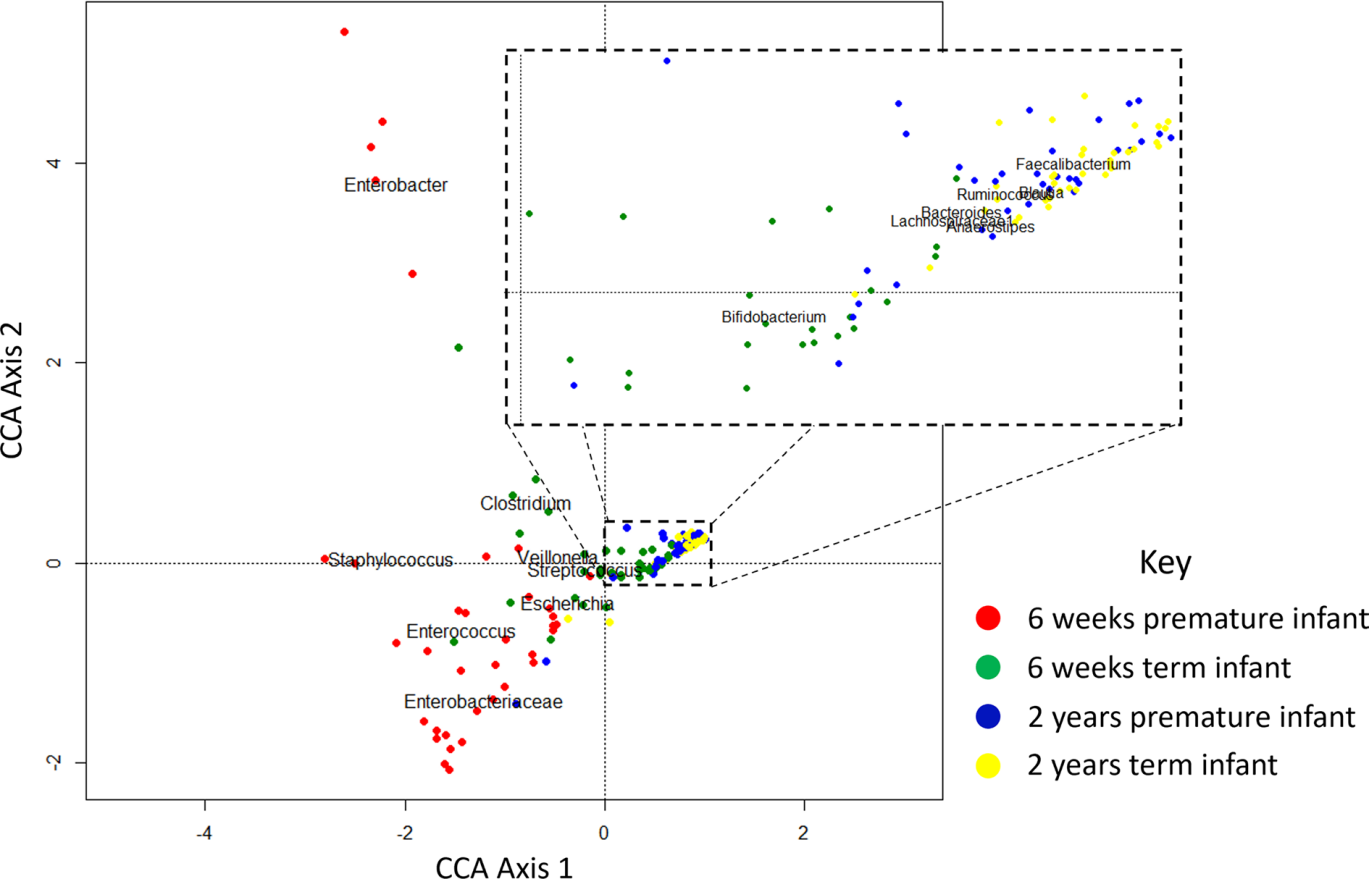
CCA showing separation of samples based on OTUs. Faecal samples taken at 6 weeks of age from premature infants are distinguished from term infants by higher abundances of Staphylococcus, Enterococcus and Enterobacteriaceae species. The microbiota of term infants feature greater abundance of Clostridium and Bifidobacterium species. By 2 years of age, the communities of both cohorts converge, with domination by genera such as Faecalibacterium, Blautia and Bacteroides

A Canonical Correspondence Analysis (CCA) of the OTUs indicates that individual bacterial groups are also driving separation between the term and premature infants (Fig. 5).

**Fig. 6:**
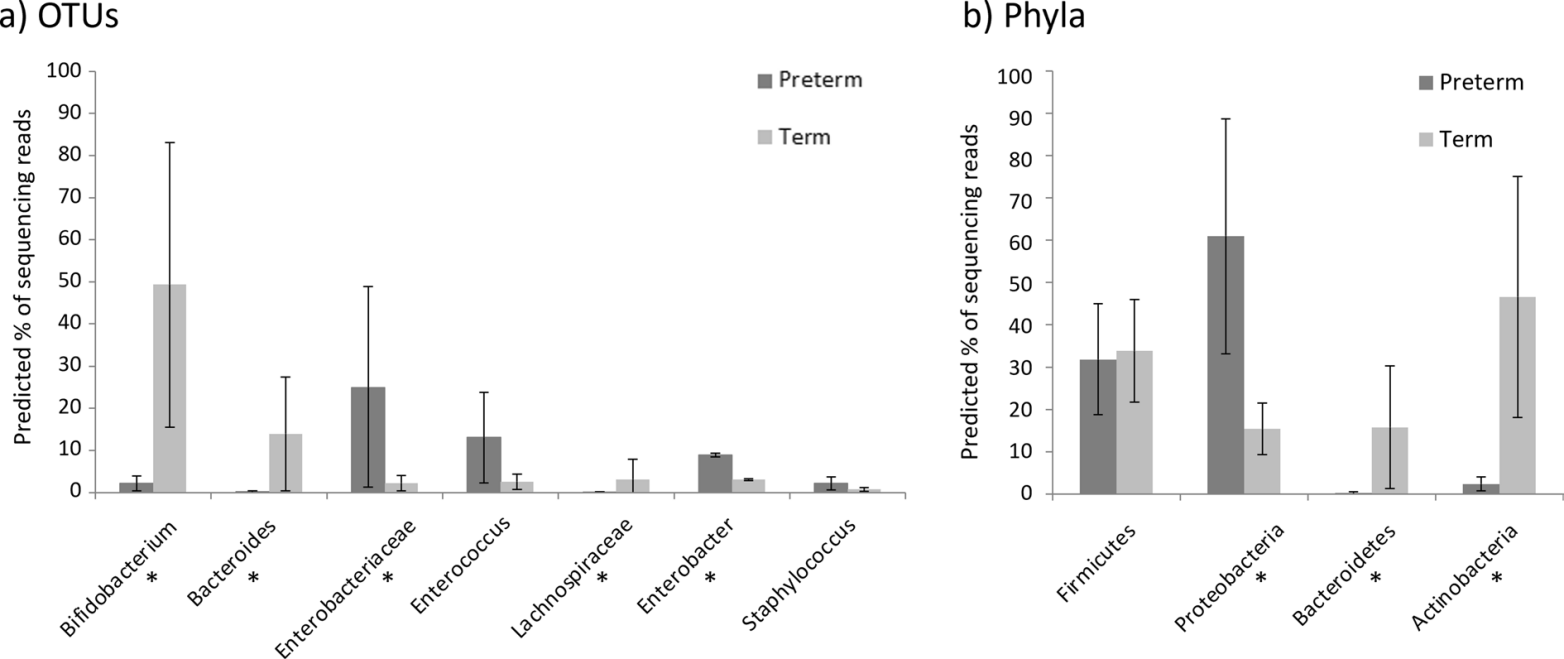
Differential abundance of **a** bacterial OTUs and **b** bacterial phyla in term and preterm infants’ faecal samples. The predicted percentage of sequencing reads (of the total in the bacterial community) for each OTU at 6 weeks of age have been calculated using the average gestational age of premature and term infants in this dataset (27.76 weeks and 40.35 weeks respectively). Predicted values are indicated by bars, with whiskers indicating the 95% confidence interval. In **a** OTUs are displayed if significantly associated with age in GLMs, whilst in **b** all phyla are shown. Asterisks indicate that the association with gestational age remained significant after MHC

To quantify the variation of specific OTUs between term and premature infants, we sought associations between gestational age and (i) the 26 most abundant OTUs (see Additional file 1: Table S1) and (ii) phyla (Additional file 1: Table S2) using GLMs. *Bifidobacterium*, *Bacteroides* and *Lachnospiraceae* were significantly positively associated with gestational age, whilst *Enterobacteriaceae* and *Enterobacter* were significantly negatively associated. These findings are reflected at the phyla level, with gestational age being significantly positively associated with Bacteroidetes and Actinobacteria and negatively associated with Proteobacteria. Model parameters were used to predict the average community structure for an average term (40.3 weeks gestation) and an average premature infant (28.1 weeks gestation) at 6 weeks of age (see Fig. 6a, b).

### Differences in the bacterial communities can be associated with specific clinical factors that differ between being born prematurely or at term

Having shown that the bacterial community is different in term compared to premature infants, we sought to determine if the differences were primarily due to prematurity itself or were more closely associated with clinical factors that differ between infants born at term and those born prematurely.

**Fig. 7:**
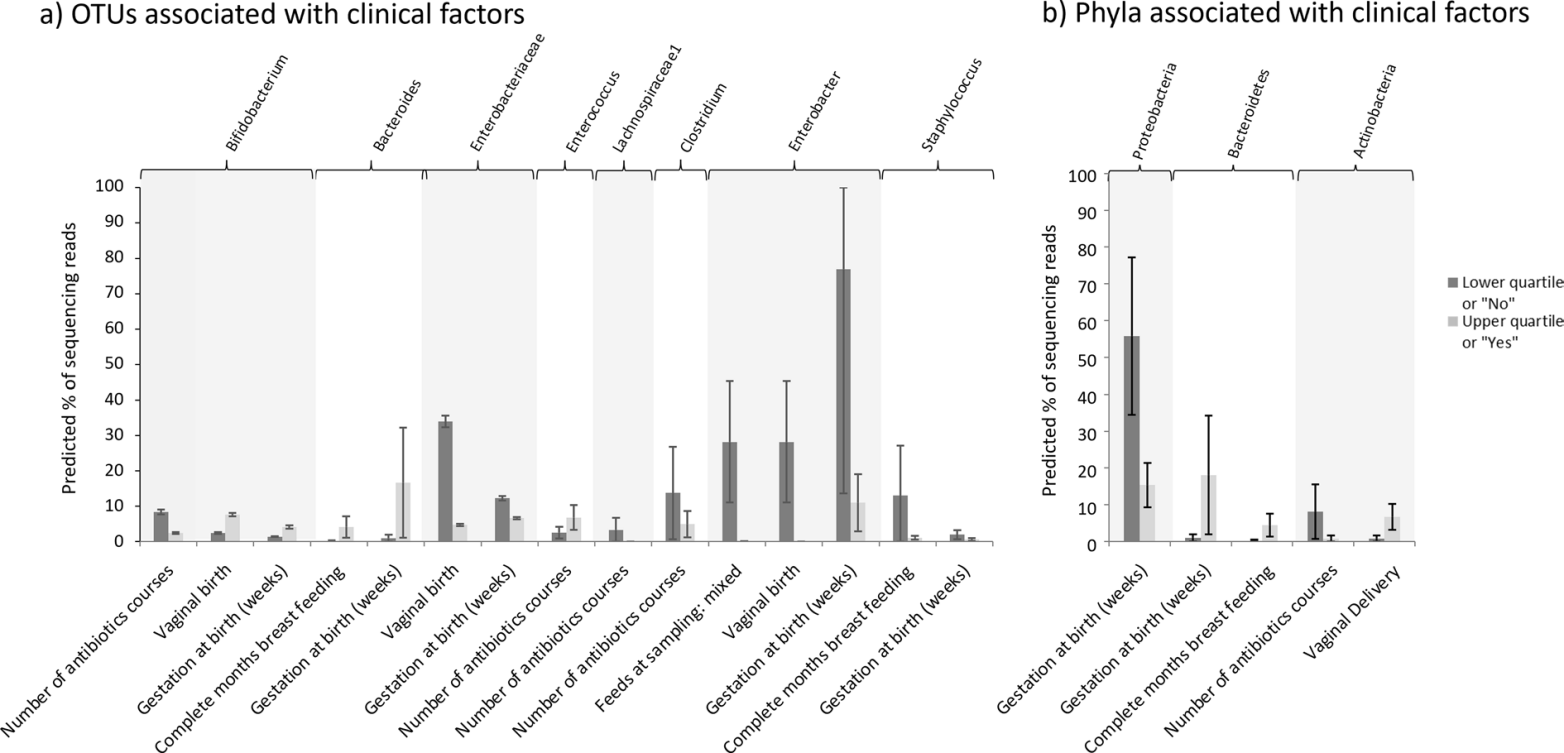
Association between **a** OTU abundance and **b** Phyla abundance and clinical factors at 6 weeks of age. Univariate and multivariate models were built where significant associations were found between clinical factors and OTU/phyla abundances. To illustrate the estimated differences in bacterial abundance when an associated clinical factor varies, the predicted percentage of sequencing reads (of the total in the bacterial community) have been calculated when a specified clinical factor is at its 25% and 75% quartile. Predicted values are indicated by bars, with whiskers indicating the 95% confidence interval. Where multiple factors were found to influence an OTU (as indicated by the top bar), each factor is illustrated separately, with either the median or the most common categorical option being used as a base value for the additional clinical factors. The effects having mixed feeds (formula and breastmilk) are compared to breastmilk feeds alone

OTUs were tested for associations with the following set of clinical factors: Feeding method at time of sampling (breast milk, formula or mixed), number of complete months of breast feeding, number of courses of antibiotics, birth method, gender, birth weight and gestation at birth (as shown in Table 1). Factors found to be significant after MHC are shown in Fig. 7a; where multiple factors were found to be associated with a single OTU, a multivariate model was used to identify dominant factors, with iterative removal of the least influential factors until only significant factors remained (see Additional file 1: Table S3). The same process was also performed with regard to phyla rather than OTUs (see Fig. 7b and Additional file 1: Table S4).

### Term and premature infant faecal microbiota converge by 2 years of age

**Fig. 8:**
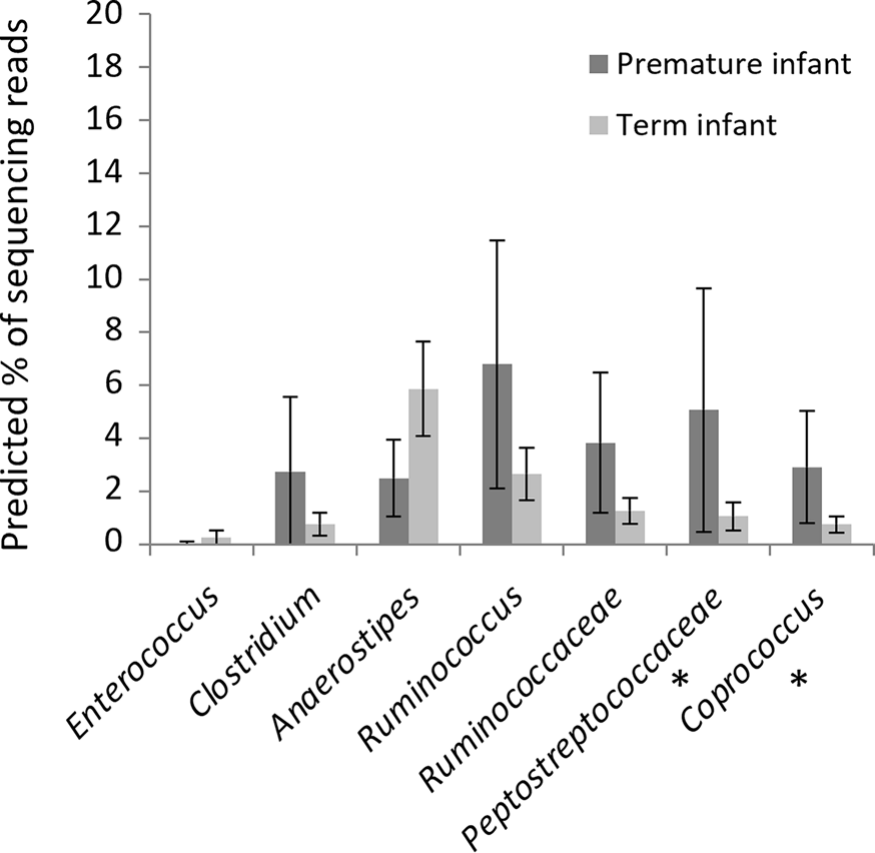
Association between **a** OTU abundance and **b** Phyla abundance and clinical factors at 2 years of age. The predicted percentage of sequencing reads (of the total in the bacterial community) for each OTU at 2 years of life have been calculated using the average gestational age of premature and term infants in this dataset (27.76 weeks and 40.35 weeks respectively). Predicted values are indicated by bars, with whiskers indicating the 95% confidence interval. OTUs are displayed if significantly associated with gestational age in GLMs. Asterisks indicate that this association remained significant after MHC

Whilst at 6 weeks of age the faecal microbiota of premature and term infants differs significantly, the microbial communities converge towards a new structure by 2 years of age (Fig. 2). Beta diversity distances between the premature and term communities are significantly reduced from the distances at 6 weeks of age (see Fig. 3), although the distances between groups are still slightly higher than the intragroup distances. The communities no longer have significant differences in terms of three alpha diversity (see Fig. 4), with both term and premature infant faecal microbiota communities increasing significantly in diversity compared to 6-week samples. Genetic diversity, as measured by Faith’s Phylogenetic Distance, is significantly higher for the pre-term cohort (p < 0.001). Only two low abundance OTUs were found to be associated with gestational age after correction for multiple tests (see Fig. 8 and Additional file 1: Table S5). No association between numbers of sequencing reads attributed to phyla and gestational age was found (see Additional file 1: Table S6).

**Fig. 9:**
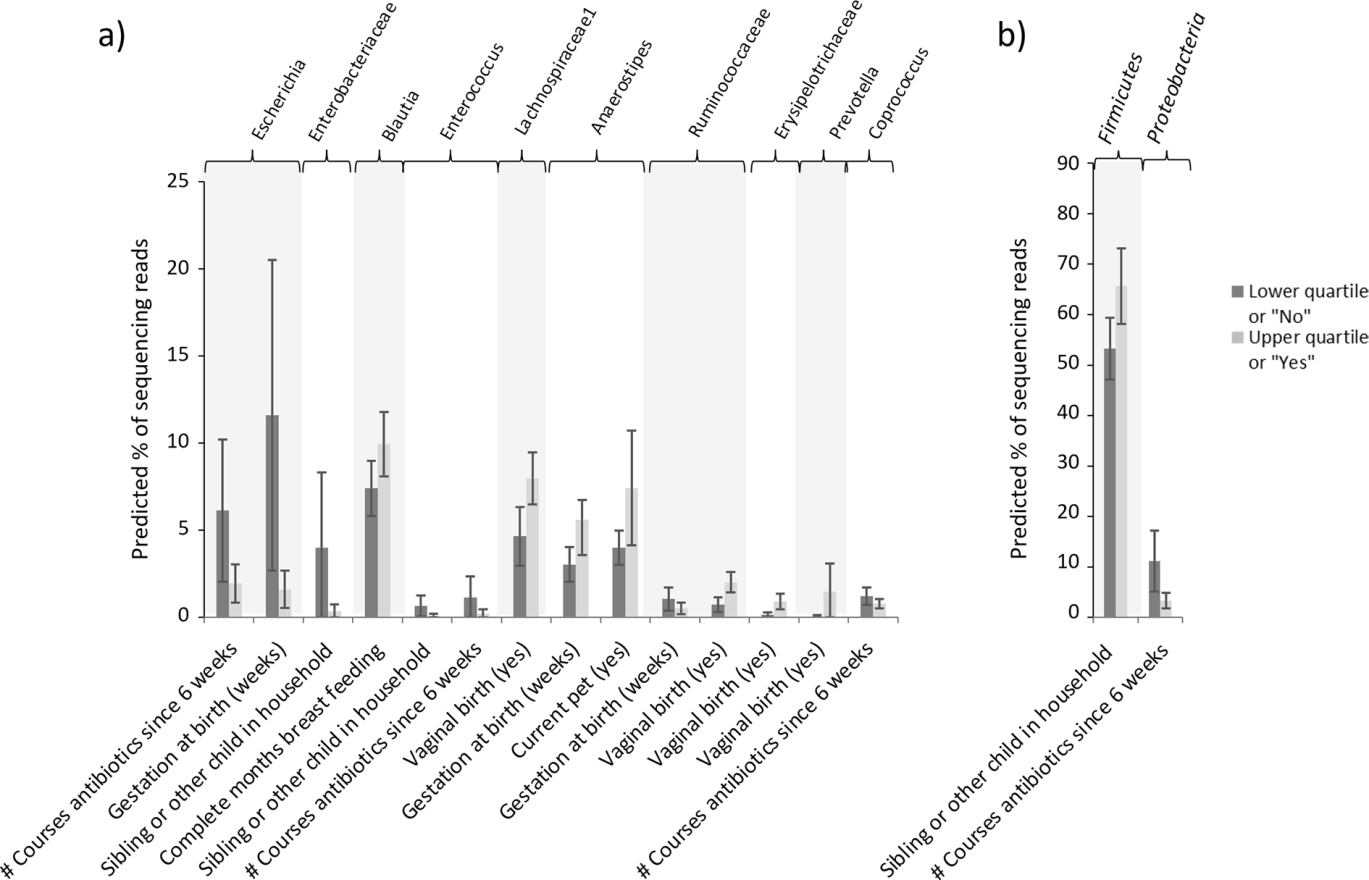
**a** OTUs and their significantly associated clinical factors at 2 years of age. **b** Phyla and their associated clinical factors at 2 years of age. Univariate and multivariate models were built where significant associations were found between clinical factors and OTU/phyla abundances. To illustrate the estimated differences in bacterial abundance when an associated clinical factor varies, the predicted percentage of sequencing reads (of the total in the bacterial community) have been calculated when a specified clinical factor is at its 25% and 75% quartile. Predicted values are indicated by bars, with whiskers indicating the 95% confidence interval. Where multiple factors were found to influence an OTU (as indicated by the top bar), each factor is illustrated separately, with either the median or the most common categorical option being used as a base value for the additional clinical factors

While there was little evidence of significant differences between term and premature infants at 2 years of age, we investigated whether elements of the microbial community at 2 years of age were associated with clinical factors (as documented in Table 1; birth demographics, 2 year demographics and number of antibiotics courses by 6 months of age). OTUs and phyla that were significantly associated with clinical factors at 2 years of age are shown in Fig. 9 (see also Additional file 1: Tables S7 and S8).

### Associations with early childhood conditions

Clinical data concerning the presence/absence of parent-reported wheezing and eczema by 2 years of age were collected for the enrolled infants. We sought to identify clinical factors and faecal bacterial signatures present both at 6 weeks and 2 years of age that were associated with the development of these two conditions. Univariate logistic regression models with a dependent variable of either wheeze (yes/no) or eczema (yes/no) were created for each set of bacterial signatures (OTUs and phyla) and clinical factors (as documented in Table 1; Birth demographics, 2 year demographics and number of antibiotics courses by 6 months of age). Premature infants are known to have a lower incidence of eczema than term infants, hence gestation was included as a confounding factor in all univariate models.

No bacterial OTU or diversity measure recorded at 6 weeks of age was found to be associated with the development either wheezing or eczema by 2 years of age. At 2 years of age, increased *Subdoligranulum* reads were significantly associated with the development of wheeze (p = 0.0018, p = 0.0468 after MHC) although the OTU represents only a small proportion of sequencing reads from the microbial community (median 1%). Increased Firmicutes were also associated with both conditions (p = 0.002 for wheezing, p = 0.007 for eczema, p = 0.007 and p = 0.029 respectively after MHC for four phyla tested). Significant associations were also found between increased weeks of birth gestation and eczema, and between increased courses of antibiotics since 6 weeks of age and the risk of wheeze (see Table 2). Factors significant to p = 0.2 after MHC were included in multivariate models to predict the odds ratios of both wheezing and eczema for infants at the median and upper and lower quartiles of each variable. Despite the relatively low contribution of *Subdoligranulum* sequencing reads to the odds ratio of increased wheezing episodes by 2 years of age, the inclusion of this factor gave a significant improvement in the model (p = 0.033).

**Table 2:** Models relating microbial and clinical data to the odds of parent-reported wheezing episodes and eczema

		Clinical factor	Odds ratio (95% CI); change in odds ratio per unit of factor	P value	Clinical factor value at:		
					Lower quartile	Median	Upper quartile
Modelling of odds ratio for wheezing episodes by 2 years of age	Univariate Models:	# Courses antibiotics since 6 weeks	**1.57** (1.19, 2.21)	0.004	0	1	3
		Bacteroidetes reads (% of community)	**0.99** (0.99, 1.00)	0.033	0	7	27
		Firmicutes reads (% of community)	**1.01** (1.00, 1.01)	0.002	44	62	72
		Subdoligranulum OTU reads	**1.05** (1.02, 1.08)	0.002	0	1	4
	Multivariate Model:	# Courses antibiotics since 6 weeks	**1.62** (1.16, 2.42)	0.010	0	1	3
		Firmicutes reads (% of community)	**1.01** (1.00, 1.01)	0.025	44	62	72
		Subdoligranulum OTU reads (% of community)	**1.03** (1.00, 1.07)	0.041	0	1	4
Modelling of odds ratios for eczema by 2 years of age	Univariate Models:	Gestation at birth (weeks)	**1.15** (1.02, 1.33)	0.030	28.1	34.4	40.3
		Firmicutes reads (% of community)	**1.01** (1.00, 1.01)	0.007	44	62	72
	Multivariate Model:	Gestation at birth (weeks)	**1.17** (1.03, 1.37)	0.030	28.1	34.4	40.3
		Firmicutes reads (% of community)	**1.01** (1.00, 1.01)	0.007	44	62	72

Univariate models included the investigated variable, infant gestational ages and day of sampling and factors have been reported if significant at the 20% level after MHC (with OTUs, phyla and clinical factors considered separate groups for correction). A multivariate model was used to identify dominant factors, with iterative removal of the least influential factors until only significant factors remained. Odds ratios (bold) and 95% confidence intervals have been calculated for each model; values provided indicate the proportional change in odds per unit of the variable

The association between gestational age and the occurrence of eczema at 2 years of age is reflected in the lack of eczema cases reported in the premature infants in this study (8 cases in 37 infants, contrasted to 24 cases in the 37 term infants). We hypothesised that this relationship may mask other signals in the data, so repeated the analysis using only data from the 37 term infants. The positive association with Firmicutes and eczema remained [p = 0.035, odds ratio of 1.01 (95% CI 1.00, 1.01)] and *Faecalibacterium* was also found to be positively associated with eczema [p = 0.021, odds ratio of 1.03 (95% CI 1.01, 1.05)] in the term cohort.

### Validation of factors associated with early childhood conditions

**Fig. 10:**
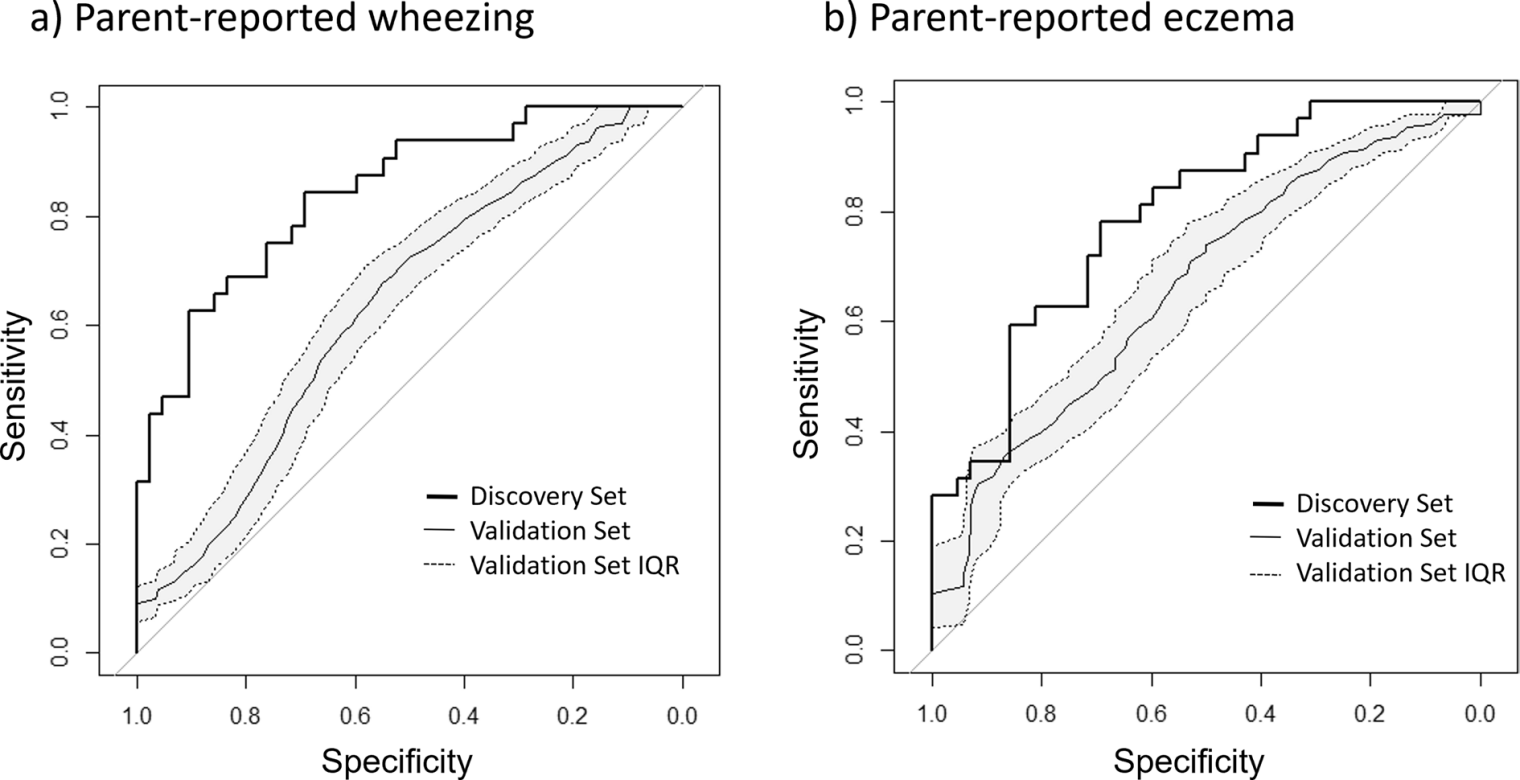
ROC curves illustrating the sensitivity and specificity of the factors identified through multivariate models. Factors identified for detecting **a** parent-reported wheeze were Firmicutes reads, Subdoligranulum reads and the number of courses of antibiotics given since 6 weeks of age and **b** parent-reported eczema were Firmicutes reads and gestation at birth. These factors were identified in the discovery set (37 matched premature and term infants), resulting in ROC curves shown in black. The validation set consisted of 30 term and 30 premature infants drawn randomly from a pool of 64 premature infants and 35 born at term 1000 times. The solid grey lines indicate the median curves from these 1000 iterations, and the dashed lines indicate the interquartile range

Given our findings that various microbial signals and clinical factors can be associated with development of eczema and wheeze, we sought to reproduce these results in a validation dataset drawn from 99 unmatched infant faecal samples at 2 years of age; 64 from infants born prematurely and 35 from infants born at term. ROC curves were created for the matched infant dataset (the discovery set) and the validation set (see Fig. 10) using the variables retained in the multivariate models (see Table 2). For the occurrence of wheeze, an area under the curve (AUC) of 0.84 was obtained for the discovery set, although this was not accurately reproduced for the validation set (AUC = 0.63, IQR: 0.61–0.66, 1000 iterations). A similar observation was found for eczema, with the discovery set having an AUC of 0.79 and the validation set an AUC of 0.66 (IQR: 0.62–0.69, 1000 iterations).

## Discussion

### Convergence of premature and term infant gut microbiota by 2 years of age

Our data demonstrate the stark difference between the microbiota of the premature infant and the term infant gut microbiota at 6 weeks of age, with significant increases in *Bifidobacterium*, *Bacteroides* and *Lachnospiraceae* in the term cohort and significantly increased *Enterobacteriaceae* and *Enterobacter* in the premature cohort. This may reflect the slower or delayed microbial succession in the premature infant gut microbiota with early dominance of facultative anaerobes and the increased use of antibiotics in premature cohorts. Arboleya et al. similarly noted a persistent, increased abundance of *Enterobacteriaceae* in premature infants up over the first 90 days of life [31]. In a term infant cohort, decreased abundances of *Bifidobacterium* and increased abundances of *Klebsiella* and *Enterococcus* have in turn been associated with antibiotic use in the first week of life, with differences persisting for 3 months [32]. We see similar associations in our cohort, with increased antibiotics course by 6 weeks of age being associated with lower abundances of *Bifidobacterium*, *Clostridium* and *Lachnospiraceae* and increased abundances of *Enterococcus*. We observed increased abundances of *Bifidobacterium* and decreased *Enterobacter* and *Enterobacteriaceae* being associated with vaginal delivery. The association of *Bifidobacterium* with vaginal delivery has been frequently observed in other studies [33–35], whilst of *Enterobacteriaceae* are less consistent, with a variety of genera associated with either delivery method. All alpha diversity measures in the premature cohort were significantly lower than the term cohort, again as observed in other studies [8, 36].

These differences are all absent at 2 years of age, indicating a convergence of all but a couple of low abundance bacterial OTUs and no significant differences between phyla are observed. In a smaller cohort, Fouhy et al. similarly observed at 2 years of age that there were no associations between phyla and gestational age at birth, with differences only observed for a small number of genera [9]. No significant differences are seen between three of four alpha diversity measures tested for the two cohorts, although the premature infant cohort now have significantly higher genetic diversity within their faecal microbiota. This could be explained by the term cohort samples being collected closer to the 2 year timepoint than the premature cohort, with a continued trajectory of increased genetic diversity being associated with increased age. Other studies have demonstrated microbial convergence by 5 years of age [37], although our data suggest this process occurs even earlier in life. Whilst there have been observations of differences in specific taxa up to 4 years of life [9], the relatively small numbers of premature infants these conclusions are drawn from is a limitation.

### Determinants of the gut microbiota at 2 years of age

Clinical factors such as delivery mode and antibiotics courses during the first 6 weeks of life are no longer associated with sizeable effects an any OTU at 2 years of age. Additional clinical factors measured since 6 weeks of age are related only to small shifts in the overall abundances of OTUs. At the phyla level, increased Firmicutes is associated with the presence of another sibling, and decreased Proteobacteria is associated with increased antibiotic treatments since 6 weeks of age. The latter could imply that blooms of Proteobacteria that are associated with antibiotic treatments may not necessarily persist. Further follow-up of these infants would be required to confirm whether there is any lasting persistence of Proteobacteria following antibiotic treatments, as this could have health consequences given the association between these bacteria and inflammatory gut conditions such as IBD [12].

### Associations with eczema at 2 years of age

Across our combined cohorts we observed an association between the development of eczema and increased Firmicutes abundance, increased gestation at birth, and, in our term infants, an association with increased abundances of *Faecalibacterium*. The association between increased gestational age at birth and risk of parental report of eczema has been observed in other cohorts [38].

There are few cross-sectional studies at a similar age to our 2-year timepoint for comparison of observed microbial associations with eczema. Zheng et al. assessed the faecal microbiota at around 1 year of age in children with parent-report of doctor-diagnosed eczema compared to controls; they did not find a difference in the phyla or diversity but found significant differences in the abundance of several genera [39]. Healthy infants had higher abundances of *Bifidobacterium*, *Streptococcus*, *Megasphaera* and *Haemophilus* in their faecal microbiota, whilst infants with eczema had increased *Veillonella*, *Lachnospiracaeae* and *Faecalibacterium*. Candela et al. (using microarrays) however found a reduction in *Faecalibacterium prausnitzii* in atopic children [40]. This apparent contradiction with our findings and that of Zheng et al. may be explained by dysbiosis at a subspecies level. Song et al. found different *Faecalibacterium prausnitzii* subspecies in faeces from atopic dermatitis patients compared to controls [41]. Other studies have not observed the association between increased Firmicutes abundance and eczema, with associations instead typically being observed at finer taxonomic resolutions. An association has however been reported between increased Firmicutes and food sensitization in infants aged 6 to 12 months in a small case control study [42]. The Firmicutes phylum does notably include the genus *Clostridium*, which has repeatedly been associated with increased risk of eczema [20, 39, 43].

### Associations between wheeze and the gut microbiota at 2 years of age

We did not find an association between the microbiota at 6 weeks and later wheeze. This may be because of the inherent problems with parent-reported wheeze affecting our case classification and the early (2 years of age) diagnosis. As we follow the cohort to school age when a more rigorous assessment of respiratory function can be undertaken, we may demonstrate an association. At 2 years of age we found a positive association between Firmicutes and *Subdoligranulum* abundances and wheeze. Whilst for most infants the number of reads for *Subdoligranulum* is low, leading to a negligible contribution to the odds ratio for wheeze, nine infants studied had 8–23% of sequencing reads in the 2-year of age sample attributed to this OTU. This result should be treated with caution given the relatively low effect size and needs further investigation, although there is a prior finding of association between sensitization to food allergens and increased faecal abundance of *Subdoligranulum* [42]. Whilst the Firmicutes phylum has not been observed to be associated with wheeze in other studies, an increased abundance of *Clostridium* has been associated with increased risk [19, 44].

### Wheeze association with antibiotic courses since 6 weeks of age

Parental report of wheeze by 2 years of age was positively associated with the number of antibiotic courses since 6 weeks of age. There are a number of factors that could explain this association which has previously been found and explored further by others [45]. There may be recall bias such that parents of children who wheezed are more likely to report antibiotic use—cross-sectional studies have found a stronger association than longitudinal studies between wheeze and antibiotic use [46]. Reverse causation may explain this association, as antibiotics may be prescribed for wheezing episodes rather than being the cause of such episodes. Other studies which have accounted for this factor have shown either no or a much smaller association between antibiotic use and wheeze [47], and in the term cohort overall accounting for antibiotics given wheeze the association became non-significant; we do not have sufficient information to conduct the same for the premature cohort but expect similar results. In addition, there may be an as yet unknown factor such as genetic predisposition to viral infections which increases the number of antibiotics prescribed and sequelae from early viral infections such as recurrent wheeze or asthma [48].

### Limitations

Whilst this study attempted to match term and premature infants, samples for terms infants at the 2-year timepoint were collected towards the beginning of their second year of life, giving a younger age on average for the term cohort compared to the premature cohort. This could have impacted some of our results, as potentially seen in our analysis of genetic diversity.

The use of 16S r RNA sequencing permitted an overview of the faecal microbiota, but lacked the resolution required to more fully investigate the associations between the microbiota and eczema given the varied effects of different *Faecalibacterium prausnitzii* subspecies in atopic dermatitis patients.

Our study relied on parental report of wheeze and eczema which is a significant limitation. Wheeze is a poorly understood term by parents which can be both underused [49] and overused [50], partly related to the first language used by parents and the age of the child. Even between medical professionals there can be discrepancy between defining a respiratory noise as wheeze [51]. Wheeze which is confirmed by a physician has been associated with higher specific airway resistance compared to that which is only reported by the parent [52]. The higher frequency of data gathering for the term cohort may have led to variation in the accuracy of recall between the two cohorts.

## Conclusion

Our data indicate a notable convergence of the premature and term infant gut microbiota by 2 years of age, with differences between the cohorts only observed for a small number of low-abundance OTUs. The microbiota at 6 weeks of age was not found to be associated with wheeze at 2 years of age, whilst development of both eczema and wheeze were associated with early life gut microbial patterns. This study benefitted from comparably large numbers of infants and identical sample processing for the two cohorts.

## Supplementary Information

**Additional file 1: Figure S1.** Sequencing read counts by sample prior to rarefaction. **Figure S2.** Shannon Diversity Index for a randomly selected 79 samples (50% of the dataset) at a range of read depths. **Table S1.** Bacterial OTUs that are significantly associated with gestational age at 6 weeks of age. **Table S2. **Associations between bacterial phyla and gestational age at 6 weeks of age. **Table S3.** OTUs and their associated clinical factors at 6 weeks of age. **Table S4.** Phyla and their associated clinical factors at 6 weeks of age. **Table S5.** Bacterial OTUs that are significantly associated with gestational age at 2 years of age. **Table S6.** Associations between bacterial phyla and gestational age at 2 years of age. **Table S7.** OTUs and their associated clinical factors at 2 years of age*. ***Table S8.** Phyla and their associated clinical factors at 2 years of age.

## Data Availability

The dataset supporting the conclusions of this article is available in the European Nucleotide Archive repository, accession number PRJEB23362, https://www.ebi.ac.uk/ena/data/view/PRJEB23362.

## Abbreviations

AUC: Area under the curve
CCA: Canonical Correspondence Analysis
CI: Confidence interval
GLM: Generalised linear models
IBD: Inflammatory bowel disease
IQR: Interquartile range
MHC: Multiple hypothesis corrections
NEC: Necrotising enterocolitis
OTU: Operational Taxonomic Unit
ROC: Receiver Operating characteristics Curve

## References

[1] Martin R, Nauta AJ, Ben Amor K, Knippels LM, Knol J, Garssen J (2010). Early life: gut microbiota and immune development in infancy. Benef Microbes.

[2] Artis D (2008). Epithelial-cell recognition of commensal bacteria and maintenance of immune homeostasis in the gut. Nat Rev Immunol.

[3] Krajmalnik-Brown R, Ilhan ZE, Kang DW, DiBaise JK (2012). Effects of gut microbes on nutrient absorption and energy regulation. Nutr Clin Pract.

[4] Shaw AG, Sim K, Randell P, Cox MJ, McClure ZE, Li MS (2015). Late-onset bloodstream infection and perturbed maturation of the gastrointestinal microbiota in premature infants. PLoS ONE.

[5] Eggesbo M, Moen B, Peddada S, Baird D, Rugtveit J, Midtvedt T (2011). Development of gut microbiota in infants not exposed to medical interventions. APMIS.

[6] Tamburini S, Shen N, Wu HC, Clemente JC (2016). The microbiome in early life: implications for health outcomes. Nat Med.

[7] Goedert JJ, Hua X, Yu G, Shi J (2014). Diversity and composition of the adult fecal microbiome associated with history of cesarean birth or appendectomy: analysis of the American Gut Project. EBioMedicine.

[8] Hallab JC, Leach ST, Zhang L, Mitchell HM, Oei J, Lui K (2013). Molecular characterization of bacterial colonization in the preterm and term infant’s intestine. Indian J Pediatr.

[9] Fouhy F, Watkins C, Hill CJ, O’Shea CA, Nagle B, Dempsey EM (2019). Perinatal factors affect the gut microbiota up to four years after birth. Nat Commun.

[10] Shaw AG, Sim K, Rose G, Wooldridge DJ, Li MS, Misra RV (2021). Premature neonatal gut microbial community patterns supporting an epithelial TLR-mediated pathway for necrotizing enterocolitis. BMC Microbiol.

[11] Neu J, Pammi M (2018). Necrotizing enterocolitis: the intestinal microbiome, metabolome and inflammatory mediators. Semin Fetal Neonatal Med.

[12] Glassner KL, Abraham BP, Quigley EMM (2020). The microbiome and inflammatory bowel disease. J Allergy Clin Immunol.

[13] Crovesy L, Masterson D, Rosado EL (2020). Profile of the gut microbiota of adults with obesity: a systematic review. Eur J Clin Nutr.

[14] Lynch SV, Boushey HA (2016). The microbiome and development of allergic disease. Curr Opin Allergy Clin Immunol.

[15] Russell SL, Gold MJ, Hartmann M, Willing BP, Thorson L, Wlodarska M (2012). Early life antibiotic-driven changes in microbiota enhance susceptibility to allergic asthma. EMBO Rep.

[16] Arrieta MC, Stiemsma LT, Dimitriu PA, Thorson L, Russell S, Yurist-Doutsch S (2015). Early infancy microbial and metabolic alterations affect risk of childhood asthma. Sci Transl Med.

[17] Abrahamsson TR, Jakobsson HE, Andersson AF, Bjorksten B, Engstrand L, Jenmalm MC (2014). Low gut microbiota diversity in early infancy precedes asthma at school age. Clin Exp Allergy.

[18] Bisgaard H, Li N, Bonnelykke K, Chawes BL, Skov T, Paludan-Muller G (2011). Reduced diversity of the intestinal microbiota during infancy is associated with increased risk of allergic disease at school age. J Allergy Clin Immunol.

[19] Stiemsma LT, Arrieta MC, Dimitriu PA, Cheng J, Thorson L, Lefebvre DL (2016). Shifts in *Lachnospira* and *Clostridium* sp. in the 3-month stool microbiome are associated with preschool age asthma. Clin Sci.

[20] Penders J, Thijs C, van den Brandt PA, Kummeling I, Snijders B, Stelma F (2007). Gut microbiota composition and development of atopic manifestations in infancy: the KOALA birth cohort study. Gut.

[21] Shaw AG, Sim K, Powell E, Cornwell E, Cramer T, McClure ZE (2016). Latitude in sample handling and storage for infant faecal microbiota studies: the elephant in the room?. Microbiome.

[22] Fierer N, Hamady M, Lauber CL, Knight R (2008). The influence of sex, handedness, and washing on the diversity of hand surface bacteria. Proc Natl Acad Sci USA.

[23] Sim K, Cox MJ, Wopereis H, Martin R, Knol J, Li MS (2012). Improved detection of bifidobacteria with optimised 16S rRNA-gene based pyrosequencing. PLoS ONE.

[24] Sim K, Shaw AG, Randell P, Cox MJ, McClure ZE, Li MS (2015). Dysbiosis anticipating necrotizing enterocolitis in very premature infants. Clin Infect Dis.

[25] Caporaso JG, Kuczynski J, Stombaugh J, Bittinger K, Bushman FD, Costello EK (2010). QIIME allows analysis of high-throughput community sequencing data. Nat Methods.

[26] Haas BJ, Gevers D, Earl AM, Feldgarden M, Ward DV, Giannoukos G (2011). Chimeric 16S rRNA sequence formation and detection in Sanger and 454-pyrosequenced PCR amplicons. Genome Res.

[27] Edgar RC (2010). Search and clustering orders of magnitude faster than BLAST. Bioinformatics.

[28] Pruesse E, Quast C, Knittel K, Fuchs BM, Ludwig W, Peplies J (2007). SILVA: a comprehensive online resource for quality checked and aligned ribosomal RNA sequence data compatible with ARB. Nucleic Acids Res.

[29] R Core Team. R: a language and environment for statistical computing. 2018. https://www.R-project.org.

[30] Oksanen JBF, Friendly M, Kindt R, Legendre P, McGlinn D, Minchin PR, O’Hara RB, Simpson GL, Solymos P, Stevens HH, Szoecs E, Wagner H. vegan: community ecology package. 2017.

[31] Arboleya S, Sanchez B, Milani C, Duranti S, Solis G, Fernandez N (2015). Intestinal microbiota development in preterm neonates and effect of perinatal antibiotics. J Pediatr.

[32] Reyman M, van Houten MA, Watson RL, Chu M, Arp K, de Waal WJ (2022). Effects of early-life antibiotics on the developing infant gut microbiome and resistome: a randomized trial. Nat Commun.

[33] Dogra S, Sakwinska O, Soh SE, Ngom-Bru C, Bruck WM, Berger B (2015). Dynamics of infant gut microbiota are influenced by delivery mode and gestational duration and are associated with subsequent adiposity. mBio.

[34] Hill CJ, Lynch DB, Murphy K, Ulaszewska M, Jeffery IB, O’Shea CA (2017). Evolution of gut microbiota composition from birth to 24 weeks in the INFANTMET Cohort. Microbiome.

[35] Reyman M, van Houten MA, van Baarle D, Bosch A, Man WH, Chu M (2019). Impact of delivery mode-associated gut microbiota dynamics on health in the first year of life. Nat Commun.

[36] Chernikova DA, Madan JC, Housman ML, Zain-Ul-Abideen M, Lundgren SN, Morrison HG (2018). The premature infant gut microbiome during the first 6 weeks of life differs based on gestational maturity at birth. Pediatr Res.

[37] Jayasinghe TN, Vatanen T, Chiavaroli V, Jayan S, McKenzie EJ, Adriaenssens E (2020). Differences in compositions of gut bacterial populations and bacteriophages in 5–11 year-olds born preterm compared to full term. Front Cell Infect Microbiol.

[38] Barbarot S, Gras-Leguen C, Colas H, Garrot E, Darmaun D, Larroque B (2013). Lower risk of atopic dermatitis among infants born extremely preterm compared with higher gestational age. Br J Dermatol.

[39] Zheng H, Liang H, Wang Y, Miao M, Shi T, Yang F (2016). Altered gut microbiota composition associated with eczema in infants. PLoS ONE.

[40] Candela M, Rampelli S, Turroni S, Severgnini M, Consolandi C, De Bellis G (2012). Unbalance of intestinal microbiota in atopic children. BMC Microbiol.

[41] Song H, Yoo Y, Hwang J, Na YC, Kim HS (2016). Faecalibacterium prausnitzii subspecies-level dysbiosis in the human gut microbiome underlying atopic dermatitis. J Allergy Clin Immunol.

[42] Chen CC, Chen KJ, Kong MS, Chang HJ, Huang JL (2016). Alterations in the gut microbiotas of children with food sensitization in early life. Pediatr Allergy Immunol.

[43] Nylund L, Satokari R, Nikkila J, Rajilic-Stojanovic M, Kalliomaki M, Isolauri E (2013). Microarray analysis reveals marked intestinal microbiota aberrancy in infants having eczema compared to healthy children in at-risk for atopic disease. BMC Microbiol.

[44] Chiu CY, Cheng ML, Chiang MH, Kuo YL, Tsai MH, Chiu CC (2019). Gut microbial-derived butyrate is inversely associated with IgE responses to allergens in childhood asthma. Pediatr Allergy Immunol.

[45] Wickens K, Pearce N, Crane J, Beasley R (1999). Antibiotic use in early childhood and the development of asthma. Clin Exp Allergy.

[46] Wickens K, Ingham T, Epton M, Pattemore P, Town I, Fishwick D (2008). The association of early life exposure to antibiotics and the development of asthma, eczema and atopy in a birth cohort: confounding or causality?. Clin Exp Allergy.

[47] Marra F, Marra CA, Richardson K, Lynd LD, Kozyrskyj A, Patrick DM (2009). Antibiotic use in children is associated with increased risk of asthma. Pediatrics.

[48] Semic-Jusufagic A, Belgrave D, Pickles A, Telcian AG, Bakhsoliani E, Sykes A (2014). Assessing the association of early life antibiotic prescription with asthma exacerbations, impaired antiviral immunity, and genetic variants in 17q21: a population-based birth cohort study. Lancet Respir Med.

[49] Shanmugam S, Nathan AM, Zaki R, Tan KE, Eg KP, Thavagnanam S (2016). Parents are poor at labelling wheeze in children: a cross-sectional study. BMC Pediatr.

[50] Elphick HE, Sherlock P, Foxall G, Simpson EJ, Shiell NA, Primhak RA (2001). Survey of respiratory sounds in infants. Arch Dis Child.

[51] Cane RS, McKenzie SA (2001). Parents’ interpretations of children’s respiratory symptoms on video. Arch Dis Child.

[52] Lowe L, Murray CS, Martin L, Deas J, Cashin E, Poletti G (2004). Reported versus confirmed wheeze and lung function in early life. Arch Dis Child.

