# Supplementary material for: Development of the gut microbiota during early life in premature and term infants
Source: Gut Pathog. 2023 Jan 16;15:3. doi: 10.1186/s13099-022-00529-6 (PMC9841687; doi:10.1186/s13099-022-00529-6)
Supplement: Supplementary file 1 — Additional file 1: Figure S1. Sequencing read counts by sample prior to rarefaction. Figure S2. Shannon Diversity Index for a randomly selected 79 samples (50% of the dataset) at a range of read depths. Table S1. Bacterial OTUs that are significantly associated with gestational age at 6 weeks of age. Table S2. Associations between bacterial phyla and gestational age at 6 weeks of age. Table S3. OTUs and their associated clinical factors at 6 weeks of age. Table S4. Phyla and their associated clinical factors at 6 weeks of age. Table S5. Bacterial OTUs that are significantly associated with gestational age at 2 years of age. Table S6. Associations between bacterial phyla and gestational age at 2 years of age. Table S7. OTUs and their associated clinical factors at 2 years of age. Table S8. Phyla and their associated clinical factors at 2 years of age. [file 13099_2022_529_MOESM1_ESM.docx]

**Additional file 1**


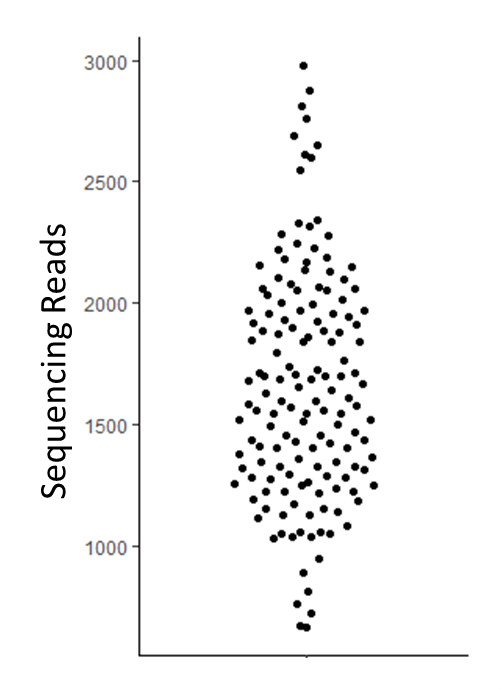


Figure S1 – Sequencing read counts by sample prior to rarefaction

***
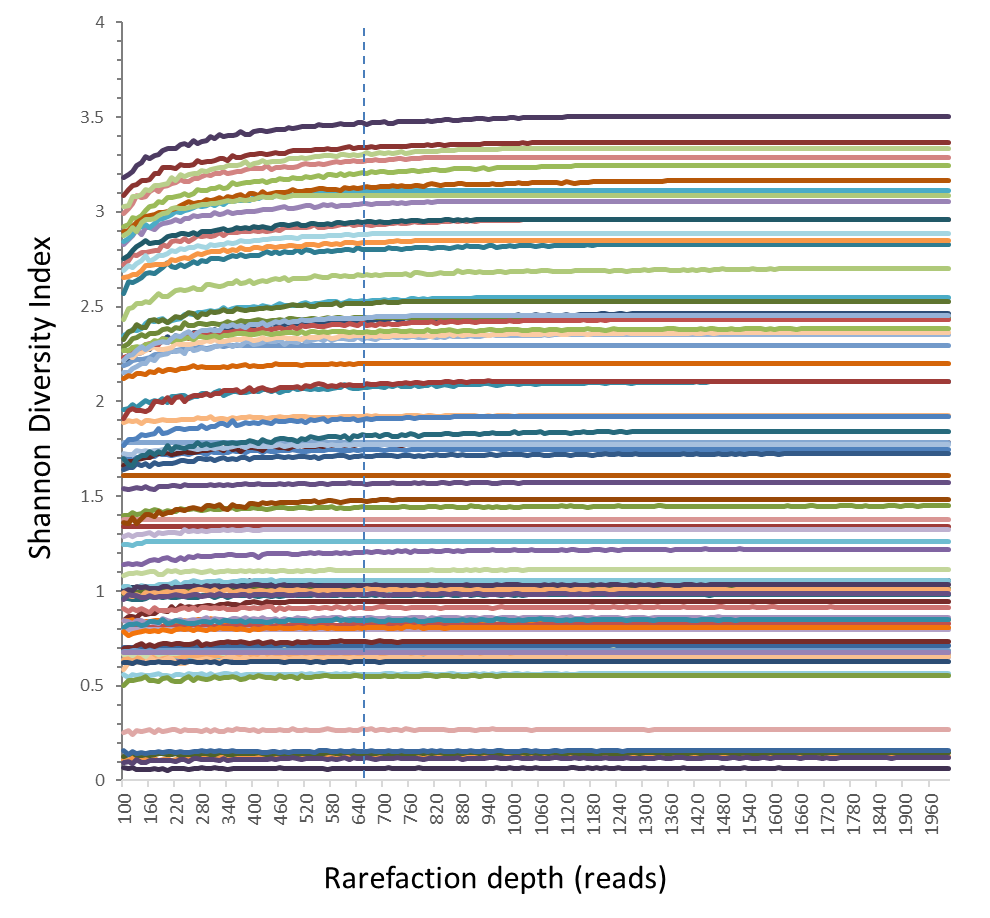
***

Figure S2- Shannon Diversity Index for a randomly selected 79 samples (50 % of the dataset) at a range of read depths. *Blue dashed line indicates the chosen read depth for rarefaction (664 reads).*

Table S1 – Bacterial OTUs that are significantly associated with gestational age at six weeks of age. *Associations were sought using general linear models with a negative binomial distribution. Day of life of sampling was included in each model as a confounding factor. The exponentiated coefficient indicates the proportional difference in an OTU for each additional week of gestational age at birth. The predicted percentage of sequencing reads (of the total in the bacterial community) for each OTU at six weeks of age have been calculated using the average gestational age of premature and term infants in this dataset (27.76 weeks and 40.35 weeks respectively). OTUs with a significant P-value prior to MHC are shown.*

Table S2 – Associations between bacterial phyla and gestational age at 6 weeks of age. *Associations were sought using general linear models with a negative binomial distribution. Day of life of sampling was included in each model as a confounding factor. The exponentiated coefficient indicates the proportional difference in an OTU for each additional week of gestational age at birth. The predicted percentage of sequencing reads (of the total in the bacterial community) for each OTU at six weeks of age have been calculated using the average gestational age of premature and term infants in this dataset (27.76 weeks and 40.35 weeks respectively).*

Table S3 – OTUs and their associated clinical factors at six weeks of age. *The coefficient of the association is shown with confidence intervals, which is exponentiated to give the proportional change (highlighted in bold) in OTU reads per unit of the clinical factor. To illustrate the effects of these shifts, predicted percentages of OTU read numbers have been calculated when the clinical factor is at its 25 % and 75 % quartile. Where multiple factors were found to influence an OTU the base values (either the median or the most common categorical option) for each clinical factor are given.*

Table S4 – Phyla and their associated clinical factors at six weeks of age. *The coefficient of the association is shown with confidence intervals, which is exponentiated to give the proportional change (highlighted in bold) in OTU reads per unit of the clinical factor. To illustrate the effects of these shifts, predicted percentages of OTU read numbers have been calculated when the clinical factor is at its 25 % and 75 % quartile. Where multiple factors were found to influence an OTU the base values (either the median or the most common categorical option) for each clinical factor are given.*

Table S5 – *Bacterial OTUs that are significantly associated with gestational age at two years of age. Associations were sought using general linear models with a negative binomial distribution. Day of life of sampling was included in each model as a confounding factor. The exponentiated coefficient indicates the proportional difference in an OTU for each additional week of gestational age at birth. The predicted percentage of sequencing reads (of the total in the bacterial community) for each OTU at two years of age have been calculated using the average gestational age of premature and term infants in this dataset (27.76 weeks and 40.35 weeks respectively). OTUs with a significant P-value prior to MHC are shown.*

Table S6 – *Associations between bacterial phyla and gestational age at two years of age. Associations were sought using general linear models with a negative binomial distribution. Day of life of sampling was included in each model as a confounding factor. The exponentiated coefficient indicates the proportional difference in an OTU for each additional week of gestational age at birth. The predicted percentage of sequencing reads (of the total in the bacterial community) for each OTU at two years of age have been calculated using the average gestational age of premature and term infants in this dataset (27.76 weeks and 40.35 weeks respectively).*

Table S7 - OTUs and their associated clinical factors at two years of age*. The coefficient of the association is shown with confidence intervals, which is exponentiated to give the proportional change (highlighted in bold) in OTU reads per unit of the clinical factor. To illustrate the effects of these shifts, predicted percentages of OTU read numbers have been calculated when the clinical factor is at its 25 % and 75 % quartile. Where multiple factors were found to influence an OTU the base values (either the median or the most common categorical option) for each clinical factor are given.*

Table S8 - Phyla and their associated clinical factors at two years of age. *The coefficient of the association is shown with confidence intervals, which is exponentiated to give the proportional change (highlighted in bold) in OTU reads per unit of the clinical factor. To illustrate the effects of these shifts, predicted percentages of OTU read numbers have been calculated when the clinical factor is at its 25 % and 75 % quartile. Where multiple factors were found to influence an OTU the base values (either the median or the most common categorical option) for each clinical factor are given.*
